# Effect of a high-fat diet and alcohol on cutaneous repair: A systematic review of murine experimental models

**DOI:** 10.1371/journal.pone.0176240

**Published:** 2017-05-11

**Authors:** Daiane Figueiredo Rosa, Mariáurea Matias Sarandy, Rômulo Dias Novaes, Sérgio Luís Pinto da Matta, Reggiani Vilela Gonçalves

**Affiliations:** 1 Department of General Biology, Federal University of Viçosa, Viçosa, Minas Gerais, Brazil; 2 Department of Cell, Tissue and Developmental Biology, Federal University of Alfenas, Alfenas, Minas Gerais, Brazil; 3 Department of Animal Biology, Federal University of Viçosa, Viçosa, Minas Gerais, Brazil; Institute for Nutritional Sciences, CHINA

## Abstract

**Background and purpose:**

Chronic alcohol intake associated with an inappropriate diet can cause lesions in multiple organs and tissues and complicate the tissue repair process. In a systematic review, we analyzed the relevance of alcohol and high fat consumption to cutaneous and repair, compared the main methodologies used and the most important parameters tested. Preclinical investigations with murine models were assessed to analyze whether the current evidence support clinical trials.

**Methods:**

The studies were selected from MEDLINE/PubMed and Scopus databases, according to Fig 1. All 15 identified articles had their data extracted. The reporting bias was investigated according to the ARRIVE (Animal Research: Reporting of in Vivo Experiments) strategy.

**Results:**

In general, animals offered a high-fat diet and alcohol showed decreased cutaneous wound closure, delayed skin contraction, chronic inflammation and incomplete re-epithelialization.

**Conclusion:**

In further studies, standardized experimental design is needed to establish comparable study groups and advance the overall knowledge background, facilitating data translatability from animal models to human clinical conditions.

## Introduction

Wound healing is a continuous and controlled process that occurs through a complex interaction between cells, extracellular matrix, blood vessels, and growth factors [1,2]. Skin repair can be organized into complementary phases such as hemostasis, inflammation, proliferation, and maturation [3–5]. Hemostasis presents as main feature interruption of hemorrhage through mechanisms such as vasoconstriction, formation of the platelet buffer and activation of blood coagulation [6,7]. The inflammatory phase consists of intense leucocyte recruitment to the wound area, removal of cellular and extracellular matrix debris, and synthesis of regulatory molecules such as cytokines and chemokine’s [8]. Macrophages and neutrophils play important roles in this phase, because are involved with the release of reactive oxygen species (ROS) and control of opportunistic infections [9]. These cells also release cytokines and chemokine’s, which attract and activated other important cells to the repair process through complex signaling pathways [8–11]. The proliferative phase progresses with an intense proliferation and migration of fibroblasts, endothelial cells, and keratinocytes; which are involved with the formation of granulation tissue rich in type III collagen, tenascin, laminin, and glycosaminoglycans, and with the progressive re-epithelialization [12–14]. During the remodeling phase, fibroblasts replace type III by type I collagen, which increases the wound tensile strength [14,15]. In addition, the number of blood vessels decreases and the wound closure is complete [16]. Finally, fibroblasts remodel the injured area and reorganize the collagen network, producing a new tissue. All phases of the wound healing process are dependent of growth factors such as vascular growth factor (VEGF), platelet-derived growth factor (PDGF) and transforming growth factor β (TGF-β), which are important in modulates cell migration, proliferation and angiogenesis [17].

It is widely known that diet and lifestyle-related factors are associated with impaired body function. Excessive consumption of high-fat foods and alcohol, for instance, are directly related to several pathological conditions, such as obesity, type 2 diabetes, insulin resistance, coronary disease, and fatty liver disease (hepatic steatosis) [11,18,19]. A high-fat diet may systematically affect the tissues, as excess fat may induce apoptosis of the hypothalamus cells and changes in homeostasis maintained by this gland [20,21,22]. Regarding the cutaneous repair processes, a high-fat diet may induce a delay in the process, which may reduce protein synthesis and leads to inflammatory and degenerative processes, as the consumption of this type of diet compromises tissue vascularization and leads to the production of free radicals [23,24].

Alcoholism is considered a public health problem due to the increased mortality rates worldwide associated with the disease [25]. Alcoholism is considered a risk factor that compromises tissue repair by increasing susceptibility to infections and reducing the number of cells of the innate and adaptive immune systems [26,27]. Its consumption is also related to the formation of ROS, which induces cell morphological and functional destruction [28]. A single exposure to alcohol can affect the proliferative phase and reduce cell multiplication and angiogenesis, as it reduces the synthesis of proangiogenic factors [29]. Another example of alcohol toxicity is the increased production of the matrix metalloproteinase MMP-8 and increased plasmin levels both could accelerate fibrin degradation in skin wounds [30,31] and reduce fibroblasts and collagen proliferation, which delay the wound- closure process [28].

Despite the increasing number of preclinical trials in the last decade, few advances have been obtained in skin repair treatments in humans. Considering that pre-clinical studies are conceived to support clinical investigations, there is a clear limitation in translating the findings obtained from wound healing in animal models to human conditions. Although some studies have shown that excessive consumption of high-fat foods and alcohol is directly related to several tissue lesions [20,28,31], we still lack knowledge on the mechanisms by which these factors retard the process of wound healing. Thus, we systematically reviewed preclinical studies that used murine models to investigate the effects of a high-fat diet and excessive alcohol consumption on cutaneous repair. We analyzed the standardized methods used in all selected preclinical studies, especially considering that the quality of the evidence generated from flawed methodological studies could compromise the generalizability of the findings and preclude the development of clinical studies.

## Material and methods

### Search for articles

The studies were selected through an advanced search on the platforms PubMed and Scopus, on October 28, 2015 (12:45:04). The expressions “animal model”, “skin tissue”, “Alcohol” and “High-Fat Diet” were used for the search on the mentioned platforms. The search for relevant studies was conducted according to the flow diagram of the Preferred Reporting Items for Systematic Reviews and Meta-Analyses (PRISMA) [32]. The researchers DRF, MMS, and RVG independently searched the PubMed and Scopus databases for all original articles about the effect of high-fat diet and alcohol on skin tissue repair in murine models. The filters were developed from the PubMed database according to the hierarchical distribution of Medical Subject Headings (MeSH Terms). A standardized search filter for animal studies was applied to the PubMed database [33]. The same search strategy was adapted and used to recover studies from the Scopus platform. The standard animal filter provided by Scopus was used. The complete search strategy is described in S1 File. Language restrictions were applied to recover only articles in English, Spanish, and Portuguese.

### Selection strategy

**Fig 1:**
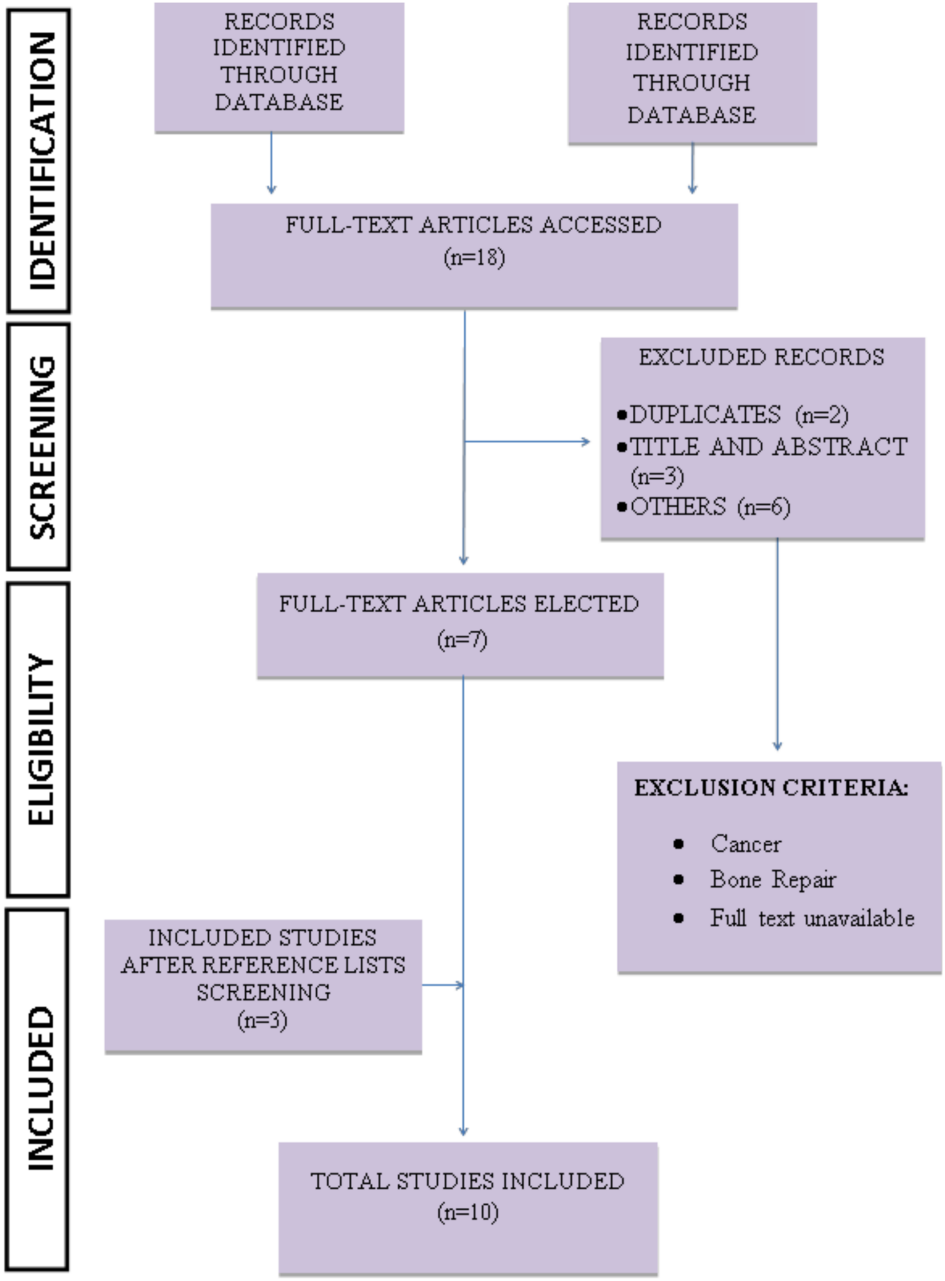
Strategy applied to recover pre-clinical studies (high-fat diet).

**Fig 2:**
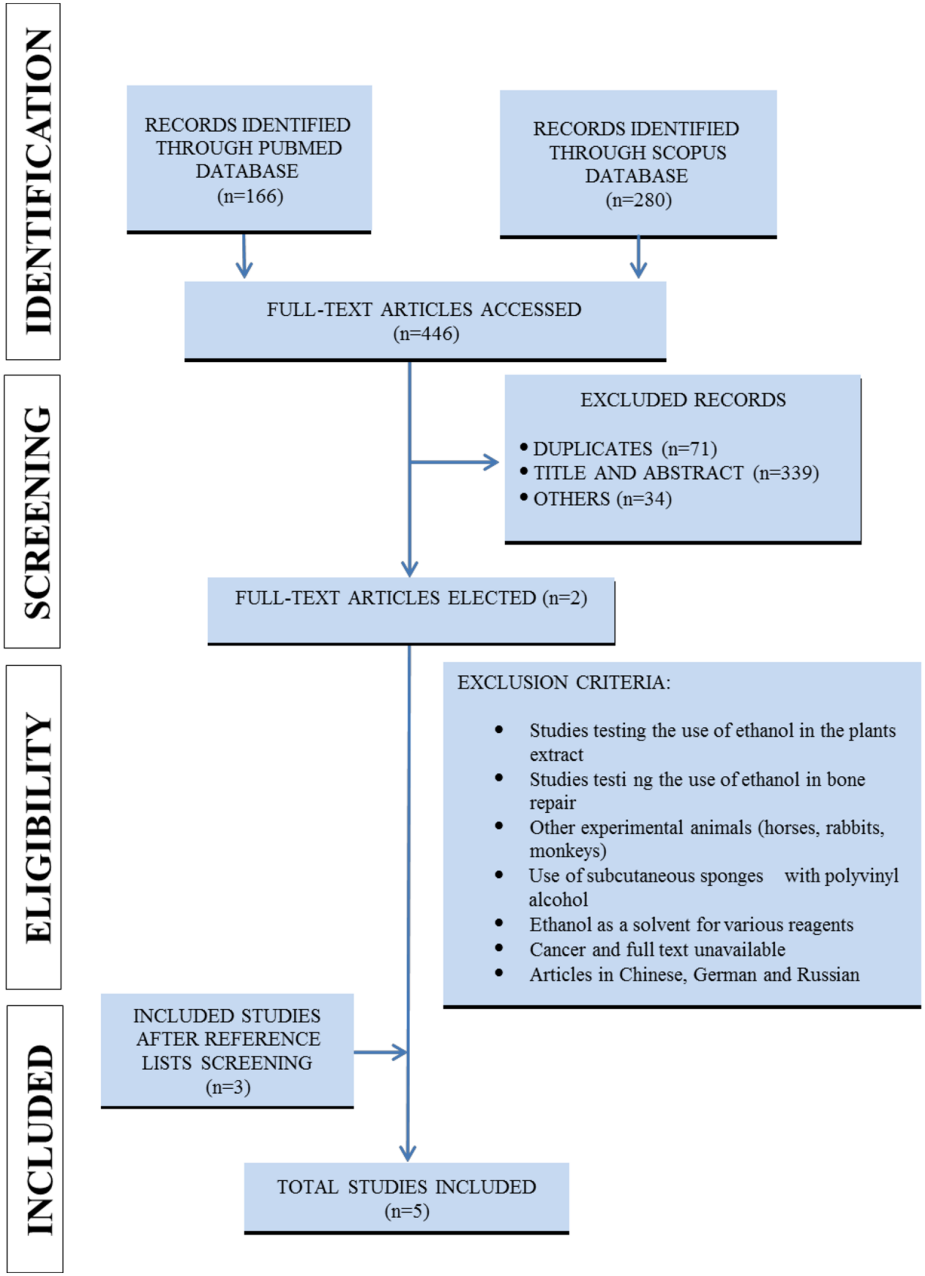
Strategy applied to recover pre-clinical studies (alcohol).

An initial selection based on title and abstract [TIAB] was independently conducted by the researchers (DRF, MMS, RDN, SLP da Matta and RVG). Duplicate studies were removed and only studies investigating the effect of a high-fat diet and alcohol on cutaneous repair in murine models were considered. After the initial search, all relevant studies were recovered in full text and evaluated according to the eligibility criteria. Studies containing the use of ethanol in plant extracts, the use of ethanol as a solvent for various reagents, other pathologies, other organs, in vitro assays, humans and other animal models, as well as secondary studies (i.e., letters to the editor, notes, reviews, and editorials) were excluded from the search (Figs 1 and 2).

### Data extraction

The following characteristics were investigated: publication characteristics (author, title, publication year, country); research methods (control group, randomization, experimental procedures, and blind evaluation of the results); experimental model (animal strain, number of animals, sex, age, weight, species, acclimatization period, animal housing, number of animals per cage and experimental group, food supply, temperature, and light cycle); characteristics of the disease model (description of the lesions), dosimetry (dose, route, and frequency and duration of the treatments); and high-fat diet composition.

### Bias analysis

Potential reporting bias was analyzed in all articles included according to the criteria described in the Animal Research: Reporting of in Vivo Experiments (ARRIVE) guidelines [34]. These criteria are based on short descriptions of essential characteristics of all studies using animal models such as theoretical and methodological basis, research objective, refinement of the analytical methods, statistical design and sample calculations and outcome measures [34]. In recent years, there has been increasing interest in systematic reviews of research involving animals. Considering that this systematic review aims to assess important aspects of the referenced publications, we built a table that summarizes all the aspects investigated, as well as their relevance, and describe positive and negative aspects of the recovered studies.

## Results

### Selected studies

Four hundred and sixty-four articles were assessed from the PubMed and Scopus databases. Seventy-three duplicated studies and 371 with inadequate thematic were excluded after their titles and abstracts were read. Twenty studies were initially selected to be included in this systematic review, and their reference lists were carefully analyzed to ensure the identification of additional relevant studies. Three studies on alcohol and three on high-fat diet were included in the systematic review after screening of the reference lists. Thus, a total of 15 studies were used in this systematic review. The complete flow diagram of the search strategy and the number of articles recovered in each step of the process is presented in the Figs 1 (high-fat diet) and 2 (alcohol) (PRISMA guidelines).

### Qualitative data

**Fig 3:**
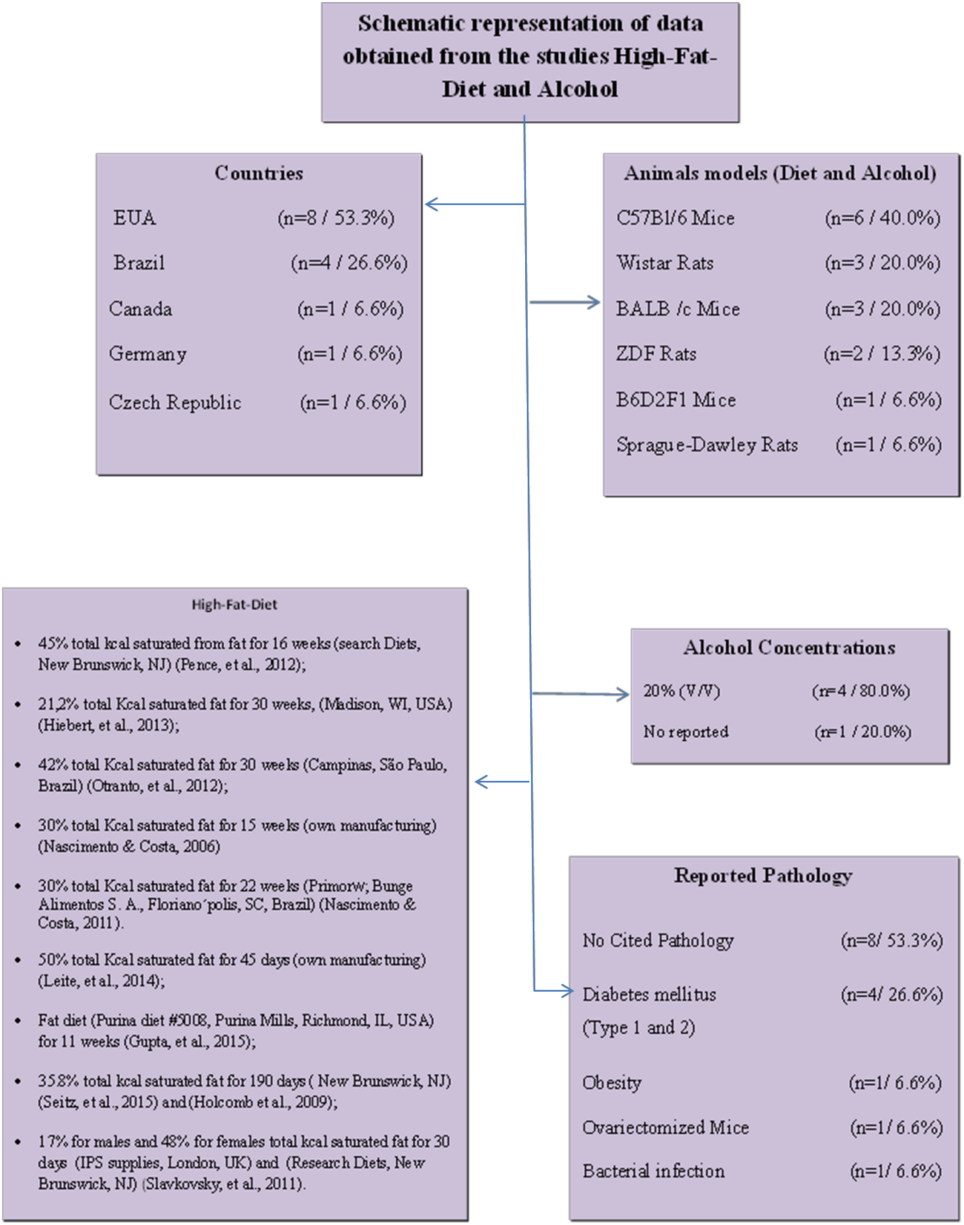
Summary of the studies describing the plants species, families, used parts of each specie, toxicity tests and popular indications.

The studies obtained were conducted in six different countries, mainly the United States of America (53.3%, n = 8), followed by Brazil (26.7%, n = 4). The remaining studies were from Canada, Germany, and the Czech Republic, with 6.6% (n = 1) each country. Rats (37.5%, n = 6), and mice (62.5%, n = 10) were the animals most frequently used as experimental models. The main mice strains cited were C57BL/6, which accounted for 37.5% (n = 6); BALB/c, 18.7% (n = 3); and B6D2F1, 6.3% (n = 1) (Fig 3). Regarding the studies using rats, Wistar rats accounted for 18,7% (n = 3); Sprague-Dawley, 6.3% (n = 1); and Zucker Diabetic Fatty (ZDF), 12.5% (n = 2). Regarding the animals gender, 46.6% of studies used only males (n = 7), 40% used only females (n = 6), and 6.6% of studies used both male and female (n = 1). Only one study did not provide this information (6.6%, n = 1). The age of the animals ranged from 6 to 37 weeks for mice and from weaning to 20 weeks for rats. This variable was neglected in 2 studies (13.3%). The body masses of the animals ranged from 17 to 50 g for mice and from 120 to 366 g for rats. The animals’ weight of was not reported in 40% of the studies (n = 6). Regarding the diet, it was observed that the higher the fat concentration, the shorter the food administration period. On average, when the diet contained 60% saturated fat, it was administered for 12 weeks, while diets with less fat (21.2% saturated fat) were administered for 30 weeks. In most studies (80.0%), the concentration of alcohol was 20% v/v (n = 4). Regarding the pathologies investigated, 26.6% (n = 4) of the studies indicated diabetes mellitus as the pathology most associated with the repair process, while 53.3% (n = 8) of the studies presented no associated pathologies (Fig 3).

Studies that mentioned that the animals were allocated to cages comprised 46.6% (n = 7) and those that did not report the animals’ housing accounted for 53.3% (n = 8). The total number of animals in the experiment was not described by 33.3% (n = 5) of the studies, and the number of animals per box was not mentioned in 86.6% (n = 13). Regarding randomization, 60.0% (n = 9) of the studies did not mention how the animals were distributed in the experimental groups. The number of animals in each experimental group was not reported in 26.6% (n = 4) of the studies. Lesion measurement intervals were reported by 86.6% (n = 13) of the studies. The anesthetics commonly used in the studies were ketamine and xylazine in 40% (n = 6) and isoflurane in 20.0% (n = 3). The food intake measurement interval of the groups treated with the high-fat diet was described in 66.6% (n = 10) of the studies (Table 1).

**Table 1:** Descriptors used for advanced search in PubMed and Scopus. Ref: References; Tox: Toxicity test; Acclim: Acclimatization period; N: number of animals per group; AB: Amount of Animals in each cage; Rand: Randomization; wk: week; d: days; M: Male; F: Female;?: not related.

**Title**	**Ref**	**Country**	**Animal Strain**	**Sex**	**Age**	**Initial weight (g)**	**Weight measurement**	**Acclim**	**Animal housing**	**N**	**Groups**	**Food Intake Measurement Interval**	**Time of diet intake**	**Related pathology**				**Rand**	**Injury**	**Lesion measurement interval**				
Exercise Speeds Cutaneous Wound Healing in High-Fat Diet-Induced Obese Mice	Pence, et al., 2012 [35]	USA	C57BL/6 mice	F	6 wk	?	1,3,5 days	1 wk	cages	5	4	every 2 wk	10 d	?				?	excisional wound 6.0-mm	Daily				
Granzyme B degrades extracellular matrix and contributes to delayed wound closure in apolipoprotein E knockout mice	Hiebert, et al., 2013 [36]	Canada	C57BL/6 mice	M	7 wk & 37 wk	?	?	?	cages	?	6	?	16 d	?				?	excisional wound 1cm	Daily				
Insulin resistance impairs cutaneous wound healing in mice	Otranto, et al., 2012 [37]	Brazil	C57BL/6 mice	M	6 wk	23–25	?	?	?	10	2	weekly	30 wk	Diabetes mellitus				Yes	excisional wound 1 cm^2^	0, 7, 14 days				
Overweight induced by high-fat diet delays rat cutaneous wound healing	Nascimento & Costa, 2006 [38]	Brazil	Wistar rat	M	?	120–150	weekly	?	cages	5 and 15	2	weekly	21 d	?				Yes	excisional wound4 cm^2^	Weekly				
Topical fentanyl stimulates healing of ischemic wounds in diabetic rats	Gupta, et al., 2015 [39]	EUA	ZDF Rats and Sprague–Dawley rats	?	11 wk	?	?	?	?	8	4	?	36 d	Diabetes mellitus				?	excisional wound 8 mm	Every two days				
Wound Healing in Mice with High-Fat Diet- or ob Gene-Induced Diabetes-Obesity Syndromes: A Comparative Study	Seitz, et al., 2015 [40]	Germany	C57Bl/6 (wild-type) and C57Bl/6 ob/ob	F	6 wk & 12 wk	15–50	?	?	cage	4	3	?	190 d	Diabetes mellitus				?	excisional wound 3-5mm	1, 3, 5, 7 and 11 days				
Zucker diabetic fatty rat: A new model of impaired cutaneous wound repair with type II diabetes mellitus and obesity	Slavkovsky, et al., 2011 [41]	Czech Republic	ZDF rats	F & M	18–20 wk	218–366	?	?	cage	Males: 9–12; females: 8–10	4	?	30 d	Diabetes mellitus				?	excisional wound 20mm	0, 2, 3 days				
Obesity Impairs Wound Healing in Ovariectomized Female Mice	Holcomb, et al., 2009 [42]	USA	C57BL/6 mice	F	6 wk	?	twice weekly	?	cage	12	3	twice weekly	23 wk	Ovariecto mized Mice				yes	excisional wound 6mm	Every two days				
Both obesity-prone and obesity-resistant rats present delayed cutaneous wound healing	Nascimento & Costa, 2011 [43]	Brasil	Wistar rat	M	After wea-ning	30 & 60	weekly	?	?	17, 16, 17	3	Daily	22 wk	Obesity				Yes	excisional wound 1 cm^2^	Weekly				
Phototherapy improves wound healing in rats subjected to high-fat diet	Leite, et al., 2015 [44]	Brazil	Wistar rat	M	?	180–200	?	?	polyethylene cages	3	4	0 and 45 day	59 d	?	Yes	excisional wound 15-mm	0, 2, 7 and 14 days							
**Title**	**Ref**	**Country**	**Animal Strain**	**Sex**	**Age**	**Initial Weight (g)**	**Tox**	**Acclim**	**Animal housing**	**N cage**	**ABh experimental group**	**Groups**	**Animal feed**	**Treatment time**	**Related pathology**	**Rand**	**Injury**	**Lesion measurement interval**	**Anesthesia (mg/Kg)**	**Dose**	**Administration (Vehicle)**			
Ethanol exacerbates T cell dysfunction after thermal injury	Choudhrya et al., 2000 [45]	USA	C57BL/6 mice	M	8–10 wk	?	?	?	?	?	?	4	?	?	?	yes	excisional wound 15% of the total body surface area	?	Sodium pentoba rbital	20% (v/v)	intraperitoneal (saline)			
Effect of Acute Ethanol Exposure on the DermalInflammatory Response After Burn Injury	Faunce et al., 2003 [46]	USA	B6D2F1 mice	M	8–10 wk	25–30	?	?	?	?	?	?	water *ad libitum*	?	Bacterial infection	?	burn wound14%-17% of the total body surface area (12 cm^2^)	?	?	20% (v/v)	intraperitoneal (saline)			
Acute ethanol exposure impairs angiogenesis and the proliferativephase of wound healing	Radek et al., 2005 [47]	USA	BALB/c mice	F	8–9 wk	17–21	?	?	?	?	4 and 6	2	?	21 d	?	?	burn wound 15% of the total body surface area	7 days	Nembutal	20% (v/v)	intraperitoneal (saline)			
Fibroblast Function and Wound Breaking Strength isImpaired by Acute Ethanol Intoxication	Ranzer et al., 2011 [48]	USA	BALB /c mice	F	6–8 wk	17 & 21	?	?	?	?	?	2	?	35 d	?	?	excisional wound 3mm	5, 7, 10, 14, 21, 28, 35 days	Ketamine—xylazine	20% (v/v)	intraperitoneal (saline)			
Effects of Acute Ethanol Exposure on the EarlyInflammatory Response After Excisional Injury	Fitzgerald et al., 2007 [49]	USA	BALB/c mice	F	8–9 wk	?	Yes	?	?	?	3 and 5	2	?	24 hs	?	?	excisional wound 3mm	6, 12, 24 hs	Nembutal	?	intraperitoneal (saline)			

### Bias analysis

The ARRIVE guidelines were used to analyze the main weaknesses of the studies. The results showed that the selected studies presented an accurate title (100.0%); abstracts containing objectives, methods, main findings and conclusions (93.3%); and introductions with sufficient scientific background (100.0%). In the experimental procedures, most studies reported the type of treatment (diet or alcohol, (100.0%), dosage (93.3%), and duration of treatment (86.6%). However, only 46.6% provided information on the animals' weight range, and only 60.0% reported the genetic modification status and previous procedures applied to the animals. Regarding housing and husbandry, 53.3% of the studies included the type of cage, number of cages, light-dark cycle, temperature, and water availability. None of the studies reported details on sample size calculation. Full details about the allocation of the animals to experimental groups, including randomization or matching, were described in 53.3% of the studies. Statistical methods used for each analysis were described in all studies, but only 66.6% specified the unit of analysis for each dataset. Only 26.6% of the studies explained the criteria for animals or data exclusion. Information about the mean and standard deviation was provided in 60.0% of the studies. Modifications in the experimental protocols aimed at reducing adverse events and information regarding the mortality of experimental animals were described in only 26.6% of the studies. Comments on study limitations, such as sources of bias, limitations of the animal model and inaccurate results were found in only 13.3% of the studies. Comments on how the findings are likely to benefit other species or systems, including relevance to human biology, were observed only in 13.3% of the articles (Table 2).

**Table 2:** Analysis of reporting bias (ARRIVE) in all included studies.

Title	Pence, 2012 [35]	Hiebert, 2013 [36]	Otranto, 2012 [37]	Nascimento, 2006 [38]	Gupta, 2015 [39]	Seitz, 2010 [40]	Slavkovsky, 2011 [41]	Holcomb, 2009 [42]	Nascimento & Costa, 2011 [43]	Leite, 2015 [44]	Choudhrya, 2000 [45]	Faunce, 2003 [46]	Radek, 2005 [47]	Ranzer, 2011 [48]	Fitzgerald, 2007. [49]	
Accurate and concise description of the article content	✓	✓	✓	✓	✓	✓	✓	✓	✓	✓	✓	✓	✓	✓	✓	100.0%
**Abstract**																
Background summary, research objectives, methods, main findings, and conclusions	✓	✓	✓	✓	✓	✓	✓	✓	✓	✓	✓	✓	✓		✓	93.3%
**Introduction**																
Sufficient scientific background	✓	✓	✓	✓	✓	✓	✓	✓	✓	✓	✓	✓	✓	✓	✓	100.0%
Explanation of the experimental approach and rationale	✓	✓	✓	✓	✓	✓	✓		✓	✓	✓	✓	✓	✓	✓	93.3%
**Objectives**																
Clear primary and second objectives	✓	✓	✓	✓	✓	✓	✓	✓	✓	✓	✓	✓	✓	✓	✓	100.0%
**Materials and Methods**																
Nature of the ethical review permissions, relevant licenses and national or institutional guidelines for the care and use of animals	✓	✓	✓	✓	✓	✓	✓	✓	✓	✓	✓	✓	✓	✓	✓	100.0%
***Study design***																
Number of animals per group	✓		✓	✓	✓	✓		✓	✓	✓			✓	✓	✓	73.3%
Information on whether the experiment was performed as a blind controlled study	✓				✓											13.3%
***Experimental procedures***																
Treatment Description (Diet or Etanol)	✓	✓	✓	✓	✓	✓	✓	✓	✓	✓	✓	✓	✓	✓	✓	100.0%
Treatment dosage	✓	✓	✓	✓	✓	✓	✓	✓	✓	✓		✓	✓	✓	✓	93.3%
Treatment Duration	✓	✓	✓	✓	✓	✓	✓	✓	✓	✓			✓	✓	✓	86.6%
Time of day for treatment administration	✓	✓	✓	✓	✓	✓	✓	✓		✓			✓	✓		73.3%
***Experimental animals***																
Information on animal species	✓	✓	✓	✓	✓	✓	✓	✓	✓	✓	✓	✓	✓	✓	✓	100.0%
Strain of the animals	✓	✓	✓	✓	✓	✓	✓	✓	✓	✓	✓	✓	✓	✓	✓	100.0%
Sex of the animals	✓	✓	✓	✓		✓	✓	✓	✓	✓	✓	✓	✓	✓	✓	93.3%
Animals' weigth range	✓			✓			✓		✓			✓	✓	✓		46.6%
Age of the animals	✓	✓	✓		✓	✓	✓	✓			✓	✓	✓	✓	✓	60.0%
Description of genetic modification status (Knock-out, transgenic, SPF)		✓			✓	✓	✓				✓	✓	✓	✓	✓	60.0%
Information related to previous procedures applied to the animals	✓				✓	✓	✓						✓	✓	✓	46.6%
***Housing and husbandry***																
Housing of experimental animals (facility type, cage or housing type, material, number of cage companions)	✓	✓		✓		✓	✓	✓	✓	✓						53.3%
Husbandry conditions (breeding program, light/dark cycle, temperature, water)			✓			✓	✓	✓	✓	✓		✓	✓			53.3%
Welfare-related assessments and interventions carried out before, during, or after the experiment	✓										✓	✓	✓	✓	✓	40.0%
***Sample size***																
Total number of animals used in each experiment and number of animals in each experimental group	✓		✓		✓	✓	✓		✓				✓	✓	✓	60.0%
Explanation for the determination of the number of animals and details of sample size calculation																0.00%
***Allocstion of animals into experimental groups***																
Full details of how the animals were allocated into experimental groups (including randomization or matching)			✓	✓				✓	✓	✓	✓		✓		✓	53.3%
Sequence of treatment and assessment of the animals in the different experimental groups									✓							6.6%
***Experimental outcomes***																
Clear experimental outcomes assessed	✓	✓	✓	✓	✓	✓	✓	✓	✓		✓	✓	✓	✓	✓	93.3%
***Statistical methods***																
Statistical methods used for each analysis	✓	✓	✓	✓	✓	✓	✓	✓	✓	✓	✓	✓	✓	✓	✓	100.0%
Unit of analysis specifications for each dataset	✓	✓	✓	✓		✓	✓		✓	✓			✓	✓		66.6%
Methods used to assess whether the data met the assumptions of the statistical approach									✓				✓	✓	✓	26.6%
**Results**																
***Baseline data***																
Description of the animals' health status, for each experimental group, before treatment	✓	✓	✓	✓		✓	✓	✓				✓				53.3%
***Number analyzed***																
Number or animals in each group included in each analysis (absolute numbers)	✓	✓	✓	✓	✓			✓					✓	✓	✓	60.0%
Animals or data not included in the analysis (and explanation for the exclusion)		✓		✓			✓						✓			26.6%
***Outcomes and estimation***																
Information (Mean = Standard Deviation)	✓	✓	✓	✓	✓	✓	✓			✓				✓		60.0%
***Adverse events***																
Information on the mortality of the experimental animals (Mean = Standard Deviation)												✓				6.6%
Modifications to the experimental protocols to reduce adverse events				✓					✓			✓			✓	26.6%
**Discussion**																
***Interpretation /scientific implications***																
Interpretation of the results, taking into account the study objectives and hypotheses, current theory and relevant studies	✓	✓	✓		✓		✓	✓	✓	✓	✓	✓	✓	✓		80.0%
Comments on the study limitations (sources of bias, limitations of the animal model, imprecision associated with the results)	✓	✓														13.3%
***Generalisability /translation***																
Comments on how the findings are likely to translate to other species or systems, including relevance to human biology												✓			✓	13.3%
***Funding***																
List of funding sources and the role of the funder(s) in the study	✓	✓	✓	✓	✓	✓	✓	✓	✓	✓	✓	✓	✓	✓	✓	100.0%
Total results	30	26	26	26	24	26	28	23	26	22	18	24	30	27	26	

✓ Means that the criteria were met according to the ARRIVE guidelines. The percentage was calculated considering the total number of studies that met the criteria included in the bias analysis

In general, the results demonstrate that high-fat diet consumption leads to delayed wound healing [35–40,42], reduced extracellular matrix components with reduced collagen synthesis [37–40,42], increased body weight [35–39,42], and high blood glucose levels [35,37,40,41,43,44]. It was also observed that alcohol consumption reduced wound healing, biomechanical strength, re-epithelialization and fibroblast proliferation [45,46,49] (Table 3). Excessive alcohol consumption reduced myeloperoxidase activity, hydroxyproline and hyaluronic acid levels [45,46,47,48,49] and chemical mediators such as tumor necrosis factor alpha (TNFα), fibroblast growth factor-2 (FGF-2), interleukin -2 (IL-2), and lysyl oxidase [46,48,49].

**Table 3:** Parameters analyzed in the studies demonstrate the effects of high-fat diet and alcohol on the cutaneous repair in murine models.

**High Fat Diet**				
**Reference**	**Wound healing rate**	**Extracellular matrix components analyzed**	**Weight**	**Glucose test**
Pence et al., 2012 [35]	Mice fed with High fat diet (HFD) showed impaired wound healing and larger wound sizes. However, wound size was significantly smaller in exercised obese mice, compared with HFD-sedentary and chow-sedentary groups	?	HFD, body weight was significantly higher, compared to the control group.	The HFD group exhibited elevated levels of blood glucose, when compared to chow-exercise, and chow-sedentary groups.
Hiebert et al., 2013 [36]	HFD exhibited reduced wound closure, delayed contraction; and chronic inflammation, compared with the control group.	The HFD-fed mice presented less collagen and decorin, and less closing wounds compared with the controls. Animals treated with the Granzime B and HFD presented increases the fibronectin and vitronectin compared with the Wild-type animals.	ApoE knouchout (AKO) mice fed a HFD presented increased weight when compared to AKO mice fed a different diet.	?
Otranto et al., 2012 [37]	The wound area was 27% greater in the high-fat chow group on day 7 and 110% greater 14 days after wounding, when compared with the standard chow (SC) group.	The collagen fibers were less organized and less dense in the HFC group. The hydroxyproline levels were lower in the high-fat chow (HFC) group.	From the 8th week, the HFC group presented higher body weight.	The blood glucose of the HFC group was higher than the standard chow (SC) group
Nascimento & Costa, 2006 [38]	21 days after wounding, the wound area in the fat diet group was 32% larger, which indicates less contraction in relation control group	Higher collagen density was observed in the control group, compared to the fat diet group.	In the fat diet group, the body weight gain was significantly greater than in the control group.	Blood concentration of glucose was not different between the groups throughout the experiment.
Gupta et al., 2015 [39]	Fentanyl treatment resulted in complete reepithelization and dense granulation tissue in the wound scars on day 36 in diabetic rats treated with high fat diet.	There was increased collagen content in fentanyl treated wounds, compared to PBS in diabetic rats treated with high fat diet.	?	?
Seitz et al., 2015 [40]	Mice fed HFD showed increased wound area and incomplete wound epithelialization in the end of the experiment, compared mice which had received a standard chow diet (CD)	?	The uptake of the HFD caused a significant increase in body weight.	The HFD group exhibited elevated levels of blood glucose.
Slavkovsky et al., 2011 [41]	Animals fed high-fat diet showed significantly increased scar size: by 40% in males, and 140% in females.	The levels of hydroxyproline, tropoelastin and procollagen were reduced in diabetic groups (HFD). MMP3 and MMP13 (matrix metalloproteinase) and MPO (myeloperoxidase) levels increased in animals fed HFD.	The animals fed HFD showed increased body weight in both sexes.The difference was more pronounced in females, twice the value of the control.	The high-fat diet animals group, presented elevated glucose levels, when compared to control.
Holcomb et al., 2009 [42]	HDF fed animals and those ovarectomized (OVX) presented 61% of the wound open, when compared to No ovarectomized (NOVX) and HFD fed mice, which presented 34% of the wound open.	?	OVX mice treated with high-fat diet presented higher final body weight than NOVX mice treated with HFD.	?
Nascimento & Costa 2011 [43]	The diet-induced obesity (DIO) group, showed no sign of reepithelialization. The percentage of the reepithelialization wound area was lower in the DIO groups compared with the control group.	In the diet-induced obesity (DIO) and diet-resistant (DR) groups, hydroxyproline levels were lower 7 days after wounding and increased slightly 14 days after wounding.	The diet-induced obesity (DIO) group presented a higher average body weight, than the diet-resistant (DR) group.	The blood glucose level of the diet-resistant (DR) group was lower than the glucose levels of the control and diet-induced obesity (DIO) groups.
Leite et al., 2015 [44]	The wound healing rate was reduced in animals treated with hyperlipidic diet and laser off.	The hydroxyproline content was reduced in the groups treated with the hyperlipidic diet and laser off.	There was no difference in the body mass of the animals after 45 days on a high-fat diet.	A higher serum glucose level, were observed in hyperlipidic animals.
**Alcohol**				
**Reference**	**Histopathological evaluation**	**ECM components analyzed**	**Cytokines**	**Weight**
Choudhrya et al., 2000 [45]	It was observed decreased proliferation of splenocytes derived from animals subjected to the combination of alcohol and burn injury.	?	A 50% decrease in IL-2 production was observed by splenocytes derived from burn animals compared with the splenocytes from sham animals	
Faunce et al., 2003 [46]	The neutrophil content of the skin of mice after burn injury was not significantly difference with of ethanol treatment, when compared to not burns (Sham+Vehicle and Sham+Ethanol) groups.	?	Myeloperoxidase (MPO) content increased in the groups Burn+Vehicle and Burn+Ethanol when compared to Sham+Vehicle and Sham+Ethanol. The production of TNF-α was lower in the Burn+Ethanol groups compared with Burn+Vehicle groups	?
Radek et al., 2005 [47]	Reepithelialization was lower in the groups treated with the Ethanol, when compared to the control groups, but does not inhibit keratinocyte migration across the wound bed.	Hydroxyproline, was significantly reduced at day 7, in wounds from ethanol-treated animals compared with the control.	The level of FGF-2 was lower in wounds from ethanol-treated mice and VEGF levels were significantly higher in wounds from ethanol-treated mice compared with the control.	?
Ranzer et al., 2011 [48]	Exposure to ethanol decreased of fibroblast proliferation, and impairment on the regulatory function of fibroblasts when compared to control.	The levels of collagen and hyaluronic acid of the wounds in mice exposed to ethanol were significantly reduced compared to control.	Lysyl Oxidase activity (LOX) in the wounds of the mice treated with ethanol decreased significantly compared to control groups.	?
Fitzgerald et al., 2007 [49]	The histological examination of wounds by myeloperoxidase (MPO) reveals a reduction at 12 and 24hs of the neutrophil infiltration. The analysis of the macrophage inflammatory protein-2 (MIP-2) reveal a reduction at 12hs, post injury in the groups treated with the ethanol	?	TNFα levels were unchanged after injury in both groups ethanol and saline-treated. IL-1β showed variables peak levels, with reduction at 6 and 12 hs and high after 24hs in the groups treated with the ethanol.	

## Discussion

A high-fat diet and excessive alcohol consumption are among the main external factors related to a lifestyle that hinders the repair process of cutaneous tissue. Tissue recovery becomes even more difficult when these two factors are associated [31,43]. Most studies included in this systematic review reported wound healing impairments following a high-fat diet and alcohol consumption. The main effects observed seem to be associated with the control of the cutaneous inflammatory and oxidative processes and the inhibition of granulation tissue formation, collagen maturation, and re-epithelialization. In this review, 461 studies were analyzed and 15 were selected as relevant after eligibility analysis. Even when only studies using murine models were included, different animal strains were observed.

The use of PRISMA guideline was essential to find all relevant papers included in the review, as it allowed the selection of different studies focused on the topic, by using a well-defined search strategy [50]. Although the difference between the species hampers the direct exploration of the applications for humans, the results of this study show the need for more strictly controlled investigations. The tissue changes induced by excessive alcohol consumption or high-fat diet intake generally manifest as a reduced wound-closure rate and morphological disorganization. The analysis of these parameters showed the great difference between the wound models, in which were realized incisions of the 3mm, until burn wound of 12 cm^2^, which exposed 15% of their total body surface area. These characteristics have direct effects on the extent of tissue morphological remodeling and mechanical strength of the scar tissue [35,36]. We must take into account that the experimental divergences such as age, duration of the treatments and diet composition, as well as dimensions of the wounds may reflect different points of interest of the researchers. Thus, it is possible that these differences were intentionally used by the authors to investigate the influence of the experimental models and treatments on the wound healing process.

The overall objective of a systematic review is mainly to develop a tool to bring together pieces of evidence from randomized experiments that evaluate the positive or negative effects of a certain intervention. This type of review has increased significantly in recent years. However, in the preclinical field, studies are still scarce and the data quality analysis of the works is still based on the criteria used in randomized clinical studies. In the present study, besides the descriptive analyses and flow diagram presenting the main findings of the papers, the ARRIVE guideline was also used to analyze the main limitations in the description and development of the selected studies. Two major limitations were observed: a large variability in the analyzed parameters and a poor specification of important details, such as animals’ age and body weight, type and size of the wounds, diet composition, and alcohol concentration. In addition, the studies reported different markers to present their findings. Mice were the most used animal model in most articles, often because of their low cost and easier husbandry, especially during long experimental periods, which generally occurs in high-fat diet models [51]. We observed that most animals were males, probably because males present lower hormonal interference than females and gain weight faster. Hormonal interference becomes more relevant when the repair process is evaluated, as it is known that female hormones have a positive effect on re-epithelialization and tissue wound closure [52,53].

Body weight ranged from 17 to 50 g in mice and from 120 to 366 g in rats. This weight range has been commonly observed in murine studies, mainly because the data are related to young and healthy animals [6,54]. Several articles did not provide the age of the animals, the number of animals per group, period of acclimatization, or light-dark cycle. The absence of important information on the methods may compromise the understanding of the study and restrain research replicability [55].

We also observed the predominance of saturated fat in the diet and discrepant time intervals between administrations. Such discrepancy may be associated with the different murine models used. This may jeopardize the development of further studies as it hampers the establishment of standardized protocols to investigate the impact of a high-fat diet on general metabolism and the secondary implications for skin wound healing. In our review, the diet administration varied from 4 to 30 weeks. Adequate tissue nutrition is essential to normal tissue repair [56,57]. In general, overweight animals present reduced wound closure and reduced matrix synthesis [20,43,44]. Considering that mice and rats present high metabolic efficiency, it is essential to develop diet models that are able to induce metabolic changes in these animals. As most studies failed to report diet characteristics and effects, it is difficult to determine to what extent the diet models used were appropriate to disrupt metabolic homeostasis and interfere in the wound-healing process.

In addition, our results showed that most works (80%) used alcohol at a concentration of 20.0% (v/v), which can be justified by well-validated models described in literature and the profile of human intake [58]. The effects of alcohol consumption on tissue destruction are well described, especially for hepatic tissue [59,60]. Considering the variability of alcohol administration used (i.e., dose, volume, frequency, and protocol duration) and the lack of a detailed protocol for the essential parameters of skin repair (i.e., cellularity, collagen, and inflammatory mediators), little information on the cutaneous repair process can be extracted. Regarding the time interval considered to measure the wounded area, total closure was not observed in most studies, once the investigators decided to adopt long intervals (0, 2, 3, 7, 14, and 21 days), instead of carrying out the measurements on the area every day. This may have been a determining factor for the results found in animals. Such studies differed from those conducted in humans, who usually present a wound closure rate of 100.0% [61]. This fact may indicate a methodological flaw, compromising the results reliability, and preventing many studies from having their results extrapolated to human conditions. The greatest flaws of the studies were disclosed in our work through the ARRIVE guideline. Open-access journals have changed the publication process by increasing research data availability. However, there is plenty of evidence that research reporting is insufficient in several research areas, which demonstrates that in many cases, even when there is high-quality science, publications do not provide enough information for replication [62]. The instructions found in the ARRIVE guideline describe the information required from all scientific publications using animals as experimental models [34]. Therefore, this may become an important tool to standardize the presentation of the methods and results.

## Conclusion

The current evidence indicates that high-fat diet and alcohol consumption delay the healing process in cutaneous tissue. Apparently, the main effects are associated with decreased stimulation of collagen synthesis and reduction of granulation tissue and re-epithelialization. Together, these effects promote a delay in wound closure and reduce the biomechanical resistance of the newly formed tissue. However, there is an urgent need to improve research reports of preclinical studies on diet and skin wound healing in murine models. This task requires the collective effort of authors, journal editors, reviewers and financial organizations to ensure the reproducibility, reliability, and generalizability of the evidence, fundamental elements to determine the exact extent of the effects of alcohol and an inappropriate diet intake on the tissue-repair process.

## Supporting information

S1 File Filters and keywords used in the search of relevant studies in PubMed and Scopus. (DOC)

S2 File PRISMA checklist. (DOCX)
